# Laid-back breastfeeding: knowledge, attitudes and practices of midwives and student midwives in Ireland

**DOI:** 10.1186/s13006-024-00619-y

**Published:** 2024-02-19

**Authors:** Margaret McGuigan, Patricia Larkin

**Affiliations:** 1 Staff Midwife, Our Lady of Lourdes Hospital, Co. Louth, Drogheda Ireland; 2 The School of Health and Science, Dundalk Institute of Technology, Co. Louth, Ireland

**Keywords:** Breastfeeding, Laid-back breastfeeding, Biological nurturing, Midwifery, Midwives, Student midwives, Ireland

## Abstract

**Background:**

Despite concerted efforts by policy developers, health professionals and lay groups, breastfeeding rates in Ireland remain one of the lowest in world, with 63.6% of mothers initiating breastfeeding at birth, dropping to 37.6% of mothers breastfeeding exclusively on hospital discharge. Nipple trauma and difficulties with baby latching are major contributors to the introduction of formula and discontinuation of breastfeeding. Research shows laid-back breastfeeding (LBBF) significantly reduces breast problems such as sore and cracked nipples, engorgement, and mastitis as well as facilitating a better latch. Although the benefits of LBBF are well documented, this position does not seem to be routinely suggested to mothers as an option when establishing breastfeeding. This study aims to determine midwives’ and student midwives’ knowledge, attitudes, and practices of using laid-back breastfeeding in Ireland.

**Method:**

A cross-sectional descriptive survey distributed to midwives and student midwives in three maternity hospitals in Ireland and two online midwifery groups based in the Republic of Ireland, during June, July, and August 2021.

**Results:**

Two hundred and fifty-three valid responses were received from nine maternity units. Most participants (81.4%) were aware of laid-back breastfeeding. However, only 6.8% of respondents cited it as the position they most frequently use. Over one-third (38.34%) had never used this position with mothers. Those more likely to suggest LBBF had personal experience of it, were lactation consultants or working towards qualification, or had participated in specific education about LBBF. Barriers included lack of education, confidence, time, and experience. Further issues related to work culture, a tendency to continue using more familiar positions and concerns about mothers’ anatomy and mothers’ unfamiliarity with LBBF.

**Conclusion:**

Although there was a high level of awareness of laid-back breastfeeding among midwives and student midwives, there are challenges preventing its use in practice. Education specifically related to using LBBF in practice is required to overcome the barriers identified. A greater understanding of mothers’ and babies’ intrinsic feeding capacities may give midwives more confidence to recommend this method as a first choice, potentially leading to more successful breastfeeding establishment and maintenance.

**Supplementary Information:**

The online version contains supplementary material available at 10.1186/s13006-024-00619-y.

## Background

Despite decades of national breastfeeding policies and action plans to improve breastfeeding rates in Ireland, data shows that considerable progress is still needed as Ireland has one of the lowest breastfeeding initiation rates in the world at 63.6%, with exclusive breastfeeding dropping to 37.6% on hospital discharge [1]. Many women that do breastfeed encounter problems, such as sore nipples and babies who have difficulty latching on [2–5]. This often leads to introduction of formula, a reduction in exclusive breastfeeding on discharge and greater breastfeeding cessation overall [3, 5].

Laid-back breastfeeding (LBBF) is an approach where the mother relaxes into a semi-reclined position with baby resting in full frontal contact, tummy down with the mother’s arms acting as guardrails instead of supporting the weight of the baby. This position enables gravity to provide positional stability for the infant, allowing up to 20 primitive neonatal reflexes to be activated, reflexes which traditional upright breastfeeding positions often inhibit [6]. It requires minimal professional input, instead using mothers’ intuitive breastfeeding skill partnered with innate infant reflex behaviours. Colson suggests that this mother baby partnership potentially empowers mothers to adopt a more active role in their breastfeeding journey [7].

Laid-back breastfeeding is a position more likely to lead to successful breastfeeding [6, 8, 9] and is recommended by many organisations, such as La Leche League International [10], the Association of Lactation Consultants of Ireland, Lactation Consultants of Great Britain, the National Childbirth Trust as well as the Health Service Executive (HSE). It is also recommended in Ireland’s National Paediatric Hospital, Our Lady’s Children’s Hospital Crumlin [11] and as part of the Baby Friendly Initiative (BFI) 20-h Breastfeeding Course [12].

In addition to reducing problems such as sore and cracked nipples and helping the infant to obtain a deeper, more stable latch this approach enables women to practice and attain skills themselves, encouraging breastfeeding self-efficacy rather than health professionals acting as experts positioning babies [13].

Following on from Colson’s seminal research [6] mainstream breastfeeding literature introduced LBBF over a decade ago [10, 14]. It then began to appear in midwifery and lactation journals [15–17]. A recent randomised control trial [8] showed this position to significantly reduce breast problems such as sore and cracked nipples, engorgement and mastitis by as much as half. A large meta-analysis from 2021 involving almost 2000 mother and infant pairs found similar results [9]. Laid-back breastfeeding is also helpful for babies with breastfeeding challenges, such as tongue-tie or receding lower jaw because gravity pulls the tongue and chin forward [18].

Laid-back breastfeeding also has advantages for midwives, such as saving time and eliminating the need for detailed latching instructions [13, 15]. Further advantages include potentially reducing job-related back, neck, and shoulder pain for health professionals from bending over to assist with breastfeeding [13], which is frequently reported by midwives as causing back pain and discomfort [19]. Midwives working in postures requiring more than five minutes bending at the waist, as in a “hands on” approach to breastfeeding help, are at increased risk of neck and upper back musculoskeletal symptoms [20].

Experts now agree that that LBBF should be the first position to offer when helping establish breastfeeding before other alternative positions are suggested [7, 10, 13]. However, anecdotal evidence suggests that despite these advantages LBBF is not often suggested to mothers and does not appear to be regularly recommended as a breastfeeding position.

Difficulties in initiating and maintaining breastfeeding are complex and multifactorial [21]. Effective breastfeeding support to build maternal confidence and skill is paramount as breastfeeding is being initiated [2, 3]. However, studies show that breastfeeding mothers in Ireland frequently do not feel adequately supported by health professionals [21–24]. The provision of safe, kind compassionate care, informed by the best available evidence is fundamental to midwifery practice [25]. National policies also emphasise the importance of using evidence based practice [26, 27]. Midwives play an integral role in helping women to initiate and sustain breastfeeding. McGuinness et al. emphasises the importance of midwives in building women’s confidence and ability to breastfeed in the early days as the key to successful breastfeeding [2]. They provide contemporary knowledge and skills enabling women to continue breastfeeding when they are discharged home. Postnatally, mothers require timely and responsive support from health professionals, who ideally have undertaken standardised breastfeeding education [23]. The HSE *Breastfeeding Action Plan 2016–2021* [27] recommends continued education and updates for Irish health professionals.

Current knowledge, attitudes, and practices of midwives in Ireland regarding LBBF have not been studied to date. Awareness of midwives’ knowledge and practices in relation to LBBF would provide important organisational information, reinforcing good practice and identifying possible educational needs. Midwifery students’ knowledge and understanding of LBBF would help identify current breastfeeding education, recognising if it is like that of current midwives or if it has developed over time. These findings have the potential to influence breastfeeding education for students and health professionals alike, increasing confidence in their practice and enabling them to empower women in their care and ultimately improve breastfeeding establishment and duration.

The practice of LBBF has yielded consistently positive results [8, 9] yet there appears to be limited knowledge about the practice in Ireland. There is a strong rationale therefore to assess midwives’ and student midwives’ knowledge, attitudes, and practice of LBBF.

## Methods aim

### Aim

To identify midwives’ and student midwives’ current knowledge, attitudes, and practices in relation to using LBBF in Ireland.

### Data collection

A cross-sectional quantitative descriptive design was used. For the purpose of the study, the term, *laid-back breastfeeding* was used rather than terms such as Biological Nurturing© [6] or “Starter position” [13] as LBBF is more familiar to midwives and student midwives.

As there were no contemporary surveys available about LBBF, a questionnaire was designed. The questions were developed by MM and PL to meet the study aims. The questionnaire included illustrations of different positions to accurately reflect participants’ preferences for specific breastfeeding positions.

The initial questionnaire was piloted by a sample of midwifery lecturers for content validity and question clarity and was adjusted in response and re-piloted. The final questionnaire consisted of 15 multiple-choice questions and one open ended comment box. See Additional file 1.

The questionnaire captured demographic data, awareness, and use of the LBBF position and asked about any specific education/training on LBBF. Questions also related to which positions were most frequently used and how confident and successful participants found using LBBF in practice. The open-ended question asked participants about perceived barriers to using LBBF. All participants remained anonymous.

### Recruitment from hospitals

Ethics—approval was granted for the conduct of the study by participating hospitals and the affiliated Educational Institutes. Three maternity units in the Republic of Ireland were approached and agreed to participate in the study over June, July and August 2021 (Table 1). The colleges associated with these units were also approached to recruit student midwives. A “gatekeeper” (link midwife) was identified in each of the clinical sites to assist with recruitment, distribution, and collection of the questionnaires. The researcher visited each clinical site on two occasions. First, to discuss the study and the optimal recruitment strategies with the gatekeeper while providing the questionnaires. The second to collect the questionnaires. In the interim, contact was made to monitor and encourage recruitment. A “SurveyMonkey” link and accompanying QR Code was made available for staff in the hospitals and for students through their institutions’ email addresses.

**Table 1:** Recruitment and sampling in participating maternity units & colleges

	**Setting**	**Sample Size**	**Annual Births**^a^
Unit A	Urban maternity hospital	188	9,000 ~
Unit B	Regional maternity hospital	99	3,000 ~
Unit C	Regional maternity hospital	40	1,400 ~
Online link	Multiple Irish maternity units	Unknown	
College #1	Midwifery Students	93	
College #2	Midwifery Students	200	

^a^National Women and Infants Health Programme— National Report 2021

### Online recruitment

Permission was given by the Irish Nurses and Midwives Organisation website manager and an Irish Midwifery e-group moderator to advertise the online version of the questionnaire to their members. A brief synopsis of the study along with the researcher’s contact details and participant information were included with the SurveyMonkey link.

Participants were asked to complete one questionnaire only. Return of the questionnaire indicated consent. A window of six weeks was given for responses to be returned. Both hard and online copies provided a brief explanation for the rationale for the study.

### Data analysis

Quantitative data from the 15 multiple-choice questions were exported and analysed using Statistical Package for Social Sciences (SPSS), version 26 [28]. Descriptive statistics were used to analyse respondents’ characteristics, present roles, educational status, registration route and length of midwifery experience. Knowledge, attitudes, and rationale for using LBBF and other positions were analysed using frequency distributions. A Likert scale was used to identify respondents’ confidence and success with the LBBF position. Analysis also included exploring associations between specific variables and demographic data.

For nominal and noncontinuous data, χ^2^ tests with Cramer’s rule for effect size were used to assess the statistical significance of associations between certain variables. Validity of the use of χ^2^ results was tested by ensuring > 80% of cells had frequencies of ≥ 5. Statistical significance was considered if *p* < 0.05 [29].

The final open-ended question asked, “*Do you think there are any barriers that prevent a midwife from using a laid-back breastfeeding position with mothers? If so, please state.”* The responses were analysed for content analysis with the assistance of NVivo software (current edition) to identify themes based on common content.

## Results

Out of a possible sample size of 327 midwives and 293 student midwives, 253 valid responses were received giving an estimated overall response rate of 40.8%. The hard copy response rate for the three main clinical sites was 24.4%, and most successful in the regional units (Unit C, 75% and Unit B, 44% of all midwifery staff). However, larger numbers of responses were received via the online questionnaire (68% of the total responses), especially from student midwives.

### Demographic data

Demographic details of respondents are outlined in Table 2. Data collected showed an almost even split of respondents between midwives and students (51% and 49% respectively).

**Table 2:** Current roles of participants

	Frequency	Percent
Staff Midwife	66	26.1
Clinical Midwife Manager	45	17.8
Student Midwife	125	49.4
Other	17	6.7
Total	253	100.0

Most respondents had qualified as midwives by undertaking or were in the process of completing the direct entry undergraduate BSc Midwifery programme (63.24%) as opposed to the Post General Nursing route. Two hundred and forty-seven (97.6%) respondents identified their place of work.

Participants had a wide range of experience. Among midwives, 52% had been qualified for 10 + years, 14% between 7–9 years, 6% between 4–6 years and 28% were qualified for less than three years.

Among student midwives, all years of education were represented including those at Internship stage and post graduate students undertaking the Higher Diploma Midwifery Programme.

### Awareness of Laid-back breastfeeding

The majority of respondents (81.4%) were aware of the LBBF position as a way to help mothers breastfeed their babies.

Awareness of LBBF was greater for midwives and students who had or were undertaking the BSc Midwifery Programme, as opposed to those who had or were undertaking a Higher Diploma postgraduate programme and becoming midwives through the post-nursing route (83.8% vs 77.4%).

Newly qualified midwives (< 3 years) were more aware of the position (92.3%) than those longer qualified (80%), which may reflect the increase of teaching/discussion of laid-back breastfeeding in lactation education over the last decade. (*n* = 128; χ^2^ = 7.84; *df 1, p* = 0.005).

Of the student midwives, 3rd and 4th year students were highly aware of LBBF as a breastfeeding position (96.8% and 91.3% respectively). Surprisingly, more than half of those completing the Higher Diploma postgraduate programme in Midwifery (53.3%) had never heard of LBBF.

Awareness of the LBBF position varied by more than 15% between clinical sites (Table 3). However, a Chi-square test for independence found a low level of evidence between awareness of LBBF and place of work. (*n* = 201; χ^2^ = 0.06; *df* 3, *p* = 0.896).

**Table 3:** Awareness of laid-back breastfeeding by clinical site

	Frequency	Percent
Unit A	79	84.0%
Unit B	62	77.5%
Unit C	30	90.9%
Other	30	75.0%
Prefer Not to Say	5	83.3%
Total	206	81.4%

### Helping to establish breastfeeding in practice

Participants were asked to choose the most frequent breastfeeding position that they tended to discuss when helping establish breastfeeding. Overwhelmingly, midwives and student midwives chose upright positions to help mothers, with 80% either using the Cradle Hold (37.6%), the Cross-Cradle Hold (28.7%) or the Rugby (under the arm) Hold (13.5%). The side lying position was less frequently suggested (13.5%) whilst only 6.8% of respondents cited the LBBF position as the position they most frequently tend to discuss when establishing breastfeeding.

These practices varied across clinical sites (Table 4). For example, a LBBF position was much more likely to be suggested in Unit B than in other hospitals – up to four times more likely than Unit A. However, while overall distribution of breastfeeding positions differed by clinical site, statistical analysis showed only a low level of evidence to suggest an association between breastfeeding position and hospital. (n-237; χ^2^ = 25.45; *p* = 0.062).

**Table 4:** Practice of breastfeeding positions taught compared with clinical site

	**Cradle Hold**	**Cross-Cradle**	**Side-Lying**	**Rugby Hold**	**Laid Back**	**Total**
Unit A	28.6%	35.2%	16.5%	16.5%	3.3%	100% [*n* = 91]
Unit B	49.3%	19.2%	9.6%	9.6%	12.3%	100% [*n* = 73]
Unit C	35.7%	28.6%	14.3%	14.3%	7.1%	100% [*n* = 28]
Other	41.0%	30.8%	15.4%	7.7%	5.1%	100% [*n* = 39]
Prefer Not to Say	16.7%	33.3%	0.0%	50.3%	0.0%	100% [*n* = 6]

Those who learned about LBBF through personal experience were almost three times more likely (18.2% vs. 6.8%) to use this position when helping mothers establish breastfeeding. (*n* = 253; χ^2^ = 8.03; *df 1, p* = 0.005).

Almost 5% of respondents were either qualified lactation consultants or midwives/students working towards qualification and may therefore be more likely to have a greater interest in and a wider experience of breastfeeding practices. Of this group 100% were aware of LBBF, with 82% having had specific training about this position which they found helpful for their practice. The remaining 18% stated that they would like to receive this education. Ninety percent of this group also found using this position successful in helping a baby to latch on. All of this group indicated that they felt ‘very’ or ‘somewhat confident’ in using a LBBF position with mothers.

Just over 44% of those who are lactation consultants or were in the process of becoming lactation consultants used it as their most frequent position when helping mothers establish breastfeeding. This is six and a half times higher than the overall respondents’ choice of this position. (*n* = 253; χ^2^ = 30.21; *df 1, p* = 0.001).

Among the remainder of respondents, frequency of using the LBBF position varied. Although most respondents were aware of LBBF, 40 of these respondents (15.8%) said that they never used LBBF when helping mothers to breastfeed. When combined with the 57 respondents who were unaware of LBBF, 38.3% of all respondents (*n* = 253) had never used this position.

When asked when they would use a LBBF position with mothers, 34% said they would use it as an addition to other positions, 13% said they would only use it when other positions do not seem helpful and 11% stated that they only tend to suggest it immediately after birth with skin-to-skin contact. Just 4% of midwives and student midwives use LBBF as their ‘go-to’ position to encourage a mother’s own skills first.

### Education and laid-back breastfeeding

Of those who had heard of LBBF, the majority had learned of it either through their midwifery education (54%) or from lactation consultants (46%). Thirty percent had heard of LBBF from other midwives, 24% from self-directed study such as conferences or reading, 17% from watching mothers and babies, 18% from personal experience of the position and 14% from breastfeeding support groups.

Eighty-five percent of midwives and student midwives had not been formally educated about LBBF. They had not received any training or lectures specifically discussing positional stability for infants and the 20 primitive neonatal reflexes that this position activates. Of the 15% of respondents who had specific education about LBBF, all found it useful for their practice. Of those who were aware of the position, but had no specific training on LBBF, 47% of respondents indicated that that they would like some educational input about it.

### Knowledge of laid-back breastfeeding

Respondents were asked to select where LBBF can be done. Laid-back breastfeeding can be done anywhere and does not require special equipment. Most respondents seemed to feel that it is a position best suited to a bed (98%) or a sofa (88%), while a fewer number (65%) chose armchair. Just over a quarter (29%) felt it could be done in public, however only 15% thought it could be done in an upright kitchen chair.

Respondents were then asked to select as many statements as they believed to be true about the environment and benefits of LBBF (Table 5). The statement selected most often as true was about allowing the baby to “head-bob” and self-attach which was selected by 82.5% of respondents. Seventy-five percent believed that using LBBF reduces back, neck and shoulder pain for the midwife. Almost 64% of respondents recognised that this is a position that can be done with the mother and baby fully clothed and 63% that it can reduce breast problems, such as sore nipples, cracked nipples and mastitis. However, just over half understood that this is a position that only requires a mother to be slightly inclined which explains why 85% of midwives and student midwives did not believe it is a position that can be done on an upright kitchen chair. Only 26.3% agreed with the statement that it resulted in greater infant weight gain.

**Table 5:** Knowledge about LBBF

True Statements	% Selected as True
**Laid-back breastfeeding…**	
*Reduces the prescriptive pattern of putting the baby to the breast and allow the baby to 'head bob' and self-attach*	*82%*
*Reduces back, neck, and shoulder pain for the midwife from bending over to assist with latching, by using a 'hands-off' approach*	*75%*
*Ensures gravity helps to keep the baby on the mother’s body, reducing the need for the mother to support the baby*	*71%*
*Can be done with mother and baby fully clothed*	*64%*
*Reduces breast problems, such as sore nipples, cracked nipples, engorgement, and mastitis*	*63%*
*Can be done with the mother only slightly reclined at a 65° angle (with 90° being completely upright)*	*55%*
*Can result in greater infant weight gain*	*27%*

### Success and confidence using laid-back breastfeeding

When asked how successful using the LBBF position had been with mothers, of those who were aware of it, 5% (*n* = 10) found it was not at all successful. The remainder who had used the position (74%, *n* = 143) found it an effective way for baby to latch by either offering encouragement, making a few adjustments, or helping attach baby to the breast in this position. Twenty one percent (*n* = 41) had never suggested this position.

Despite almost 11% of respondents reporting feeling very confident using this position and over half (54.6%) feeling somewhat confident, the position was still not often suggested when helping mothers establish breastfeeding. Just over 30% of respondents reported they were ‘not very’ or ‘not at all confident’ using LBBF with mothers (Table 6).

**Table 6:** How confident do you feel using the laid-back breastfeeding position with mothers?

		Frequency	Percent
Valid	Very	21	10.8
	Somewhat	106	54.6
	Not very	48	24.8
	Not at all	13	6.7
	Not applicable	6	3.1
Total		194	100.0

### Midwifery education specific to lbbf and its effects in practice

Those who had received LBBF training were nine times more likely to cite LBBF as the position they most frequently used compared to those with no training (27% vs 3%), with a significant correlation shown. (*n* = 181; χ^2^ = 20.59; *df 1, p* = 0.008). A large proportion of participants (78.8%) who have not had or are unsure if they have had specific training about LBBF had never used the position with mothers and babies.

For those participants who said that LBBF was their “go-to” position for helping mothers the results showed that they were represented in all roles, including student midwives. However, only 1.8% of respondents who did not have training cited using LBFF as their “go-to” position compared with 22% of those who did. (*n* = 196; χ^2^ = 21.21; *df 1, p* = 0.001).

Of those who had been given specific education about LBBF, 94.3% found using this position successful. However, even for those without this education 70% reported using LBBF successful, which suggests how well the position works when babies are given the chance to latch on in this manner.

However, midwives and student midwives who did receive specific training about LBBF were more confident using this position than those who hadn’t, with 95% either very or somewhat confident, compared to 58% of those who had not received training reporting the same level of confidence (Table 7), with a high degree of association between these two variables. (*n* = 196; χ^2^ = 21.12; *df 1, p* = 0.001).

**Table 7:** Midwifery education specific to LBBF and effects in practice

	Had LBBF Education	No LBBF Education
LBBF as their “go-to” position	22%	1.8%
Very or somewhat confident using LBBF	95%	58%
Found LBBF successful when used	94.3%	70%
Have never tried LBBF with mothers	0%	78.8%

### Barriers to using laid-back breastfeeding

**Fig. 1:**
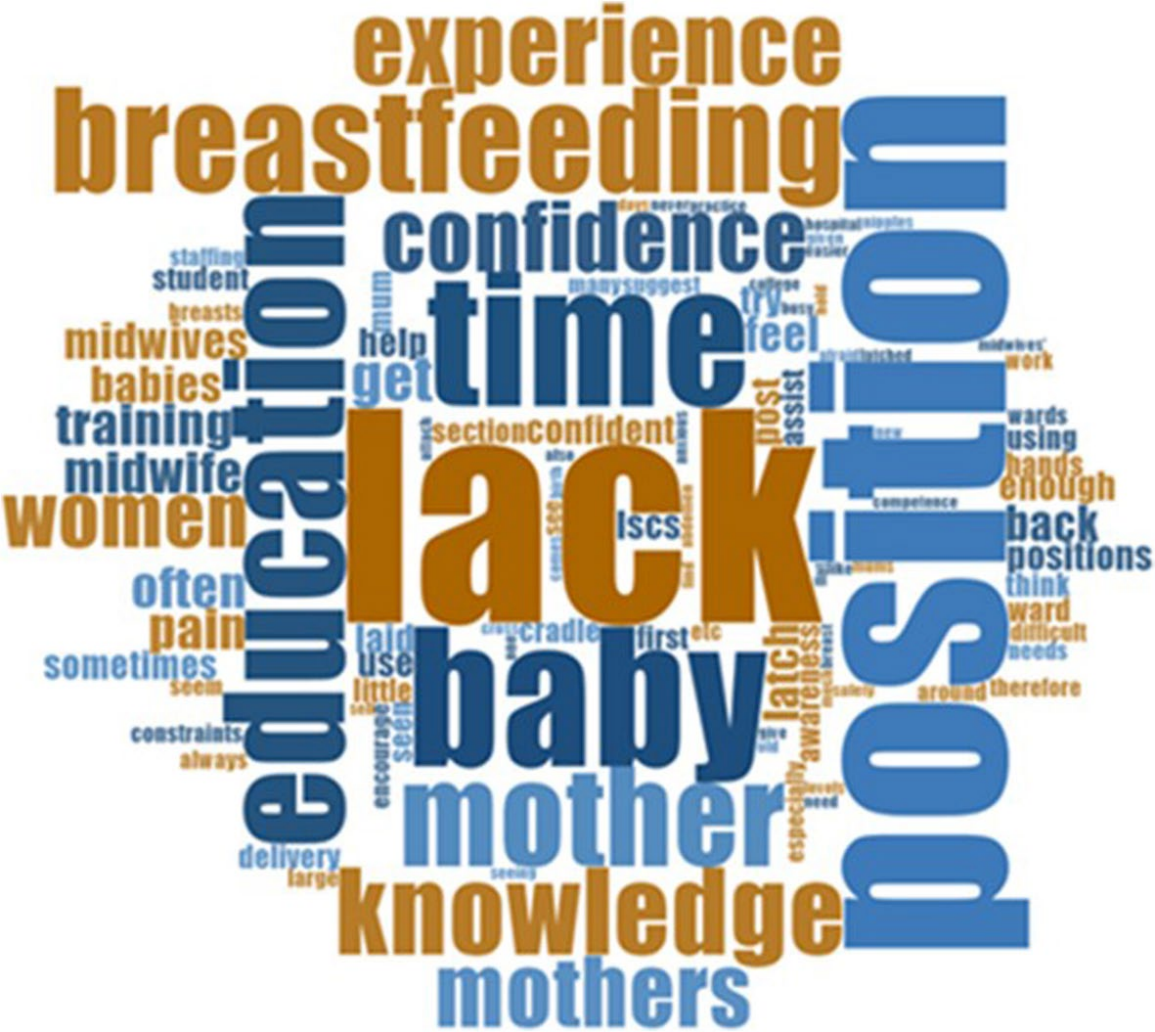
Main themes related to barriers to using LBBF

Fifty-six percent of all respondents (*n* = 110) who were aware of LBBF submitted an open-ended response about any perceived barriers to using a LBBF position. Seven common themes were identified, mostly citing a “lack of” something (Fig. 1). See Table 8 for respondents’ quotes on this question.

**Table 8:** Quotes from replies to the question: “Do you think there are any barriers that prevent a midwife from using a laid-back breastfeeding position with mothers? If so, please state”

THEME	QUOTE	ROLE
LACK OF EDUCATION/KNOWLEDGE/TRAINING	*“No training given on this position.”*	Staff Midwife, 0–3 years qualified
	*“My college education was lacking on breastfeeding. Everything I know and feel confident in comes from personal experience of breastfeeding or outsourced education. I don’t think there’s enough breastfeeding education in college.”*	Midwifery Student Intern
	*“Lack of education/training. Not one of the ‘traditional’ positions taught.”*	Clinical Midwife Manager, 10 + years qualified
LACK OF TIME/STAFF SHORTAGE	*“It takes time for a baby to latch by themselves in this position in my experience. Often, we are under time pressure to make sure that baby has had a first feed (I work in delivery) and so you are more hands on in order to ensure that this has happened.”*	Staff Midwife, 0–3 years qualified
	*“Time! On delivery suite there is very little time to aid the establishment of breastfeeding, and often following the birth experiences women have there, there is little time to try more than one position. The attitude is “get the baby on the breast and get the woman out of here” which is very sad and frustrating as a midwife”*	Clinical Midwife
	*“Breastfeeding in general can be difficult to support on busy/understaffed wards, which often do not seem to encourage breastfeeding.”*	Manager, 7–9 years qualified2nd year Student Midwife
LACK OF EXPERIENCE/CONFIDENCE	*“Personally, I have very little experience with this position and wouldn’t feel confident suggesting it to mothers.”*	3rd year Student Midwife
	*“Midwives’ confidence using this position (is a barrier).”*	Clinical Midwife Manager, 7–9 years qualified
LACK OF AWARENESS/POPULARITY	*“It’s not the ‘norm’ – you don’t see it often, so you don’t think to suggest it.”*	2nd year Student Midwife
	*“Not in the media/not what women see on TV, etc.”*	Staff Midwife, 10 + years qualified
CULTURAL/TRADITIONAL PRACTICES	*“Remaining as hands off as possible” (is difficult)*	Staff Midwife, 7–9 years qualified
	*“Easier for mum to attach, but harder for midwife to assist baby with latch.”*	Staff Midwife, 10 + years qualified
OTHER INFLUENCES	*“C-section. I often had women say the babies’ feet were hurting them post incision.”*	3rd year Student Midwife
	*“Pain after LSCS/Instrumental can impact positioning and also lead to greater tiredness.”*	Staff Midwife, 0–3 years qualified
	*“I am a student and my preceptors have never mentioned this position. As a student we don’t feel we have the authority to suggest a “new” technique to mothers.”*	1st year Student Midwife
MATERNAL ISSUES	*“Mother’s hesitation.”* *“Large breasts.”* *“Many mothers are afraid of it.”* *“Mother’s unwillingness.”* *“Not confident if the baby can breathe (mother worries). Is the mother confident in this particular position?”* *“Lack of awareness on the part of the mother.”*	Midwifery Students
	*“Mothers are unfamiliar with it.”* *“Baby not willing to latch, mother’s flat nipples, anxious mother.”* *“Mum’s anatomy (inverted/flat nipples or large breasts) requires other positions which are easier for baby to latch on.”* *“Mums’ lack of knowledge of this breastfeeding position.”* *“Anxious mothers.”* *“Mothers’ confidence in their own bodies and handling baby.”* *“Some mothers are afraid of not seeing what the baby is doing, especially if the mother has larger breasts.”*	Midwives

Lack of time to try a position that may take longer, lack of staff, lack of education/training, lack of knowledge, lack of experience and lack of confidence suggesting this position, and lack of awareness/popularity of it were the main reasons offered as barriers.

Many respondents recognised that reluctance to use LBBF stemmed from historic cultural practices and traditional midwifery teaching to use “hands on” techniques.

LBBF was approached with some trepidation and with concerns about safety issues. Some respondents believed babies may be unstable and could fall or perceived that newborns were too young to feed in this position. Others felt lack of equipment hindered them from using LBBF. However, frequently the barrier suggested by midwives and student midwives to using LBBF were concerns about the mother herself, regarding her anatomy, her emotional state, or her unfamiliarity with the position as a reason they would not suggest LBBF.

### Lack of education/knowledge/training

Overwhelmingly, midwives and students mentioned their lack of knowledge about the LBBF position as the biggest reason why they are not comfortable suggesting it. The words “lack of training or education” were mentioned in 28% of the 110 responses to the open-ended question.

Some felt that overall education on breastfeeding for midwives was not enough.

### Lack of time/staff shortage

Another common reason for not suggesting LBBF was due to time pressure. Midwives and students frequently mentioned that they didn’t have time to help mothers as they would have liked.

Respondents perceived that the LBBF position takes longer for baby to latch, and it was easier and quicker for the midwife to physically latch the baby to the mother’s breast. Emphasis on pressure to establish the first feed in the labour ward was evident.

Time constraints and under staffing also contributed to a lack of breastfeeding in general for new mothers.

### Lack of experience/confidence

In 21% of the total 110 responses, participants suggested that their own lack of experience using this position with mothers led to a lack of confidence in recommending it.

### Lack of awareness/popularity

Respondents also recognised that LBBF isn’t often suggested because they just don’t see it used in practice and therefore don’t think to use it.

### Cultural/traditional practices

Midwives were very candid in acknowledging that they may not use LBBF with mothers because they were more comfortable to continue using ‘hands on’ positions and found it difficult to revert to a more ‘hands off’ approach.

### Other influences

Some participants were influenced by concerns about mothers’ comfort, suggesting that LBBF may be uncomfortable for mothers who may be in pain from an instrumental birth or Caesarean section. However, some midwives felt LBBF is the best position to use for mothers post Caesarean section.

### Maternal issues

Twenty-eight percent of the open-ended responses cited specific issues with the mother or her baby as a reason for not using a LBBF position. For the mother, issues such as her anatomy (large breasts, flat/inverted nipples, raised BMI), her anxiety, her lack of understanding or her unwillingness to try something new were mentioned. For the baby it was difficulties with latch and/or a sleepy baby.

### Equipment/environment issues

Some midwives felt that the hospital environment itself or poor equipment made it difficult to suggest LBBF to mothers. These included lack of pillows, ward layout and lack of bedspace. Some midwives felt lack of available chairs at the beside was a barrier while others thought that they couldn’t suggest LBBF if a mother was seated in a chair at the bedside.

### Other issues

A number of respondents felt it was a position better suited to older babies and not easy to get newborns to feed in this position. Some respondents expressed concerns about the safety of using this position, regarding the stability of the baby, especially when mothers were tired.

Students found it challenging to apply their knowledge of using a LBBF position to the clinical area when it was not already being practiced there.

## Discussion

This study aimed to discover the current awareness, knowledge, and practices of midwives and student midwives regarding LBBF in Irish hospitals.

Despite high awareness of LBBF (81.4%), application of this position when helping establish breastfeeding was rarely evident in practice, with only 6.8% of midwives and midwifery students using it as their most frequent position to try with mothers. Significantly, almost 40% of midwives and student midwives surveyed have never used this position at all.

Midwifery students expressed a lack of confidence to challenge cultural norms about teaching breastfeeding, especially the current hands-on approach and did not feel comfortable using a different technique than their preceptor. Recent UK research by Spencer [30] found that students experience predominantly negative attitudes about breastfeeding from their preceptors in clinical practice, which would make discussions difficult. Interestingly, more than half of postgraduate students had never heard of LBBF and none of them used it as their “go-to” position, which may indicate a need for reassessment of this part of their breastfeeding education.

For midwives, it was the more newly qualified staff (< 3 years) that had the most awareness of the position (92.3%), which may reflect the increase of teaching/discussion of LBBF in lactation education over the last decade. However, only 5.3% of midwives working on the wards used this position in practice most often. So, despite an even higher awareness of LBBF than the total number of respondents, there is even less practice of it among staff midwives. This may suggest a hesitancy to challenge current practice.

Laid-back breastfeeding was used by most midwives as an alternative to more familiar positions. As LBBF is relatively new in midwifery education [31] respondents are more attuned to what the literature terms as ‘traditional’ positions that mainstream breastfeeding education adopts [15, 32]. Very few midwives suggest LBBF as their first choice, preferring to use it as a reserve for times when other positions are not working or as an addition to upright positions.

The more popular upright breastfeeding positions are more likely to lead to a shallower latch, contributing to problems such as nipple pain [8, 9, 15, 16]. An Australian study involving 653 mother-infant pairs found that mothers who used the cross-cradle hold in particular were four times more likely to experience nipple pain and trauma [33]. In Unit A this was the most frequently used position. The availability of LBBF as an option could potentially help alleviate such common issues.

Education specific to LBBF could provide midwives with an understanding of how and why a laid-back position enables babies’ primitive neonatal reflexes to stimulate breastfeeding and how upright positions can hinder successful breastfeeding [6]. This can address commonly held misconceptions regarding the superiority of upright positions. Enabling a baby driven approach can lead to greater breastfeeding self-efficacy of mothers. Midwives enhancing mothers’ confidence in their own abilities may ultimately be more important to breastfeeding success than the clinical aspects of teaching [13, 34, 35].

Laid-back breastfeeding can address issues encountered with traditional breastfeeding positions used by the majority of respondents (Table 4) which can be tiring and uncomfortable for both mother and midwife [13, 19, 20]. Respondents suggested that lack of equipment, such as stools, chairs and pillows were barriers to using a LBBF position. However, education will highlight that extra equipment is not needed for LBBF. Full frontal contact and positional stability enabled by LBBF uses gravity to secure the infant’s body to the mother’s eliminating the need for props such as cushions, wrap around pillows and footstools to keep the baby at the correct height [6, 13, 15].

It is noteworthy that nearly 45% of respondents were unaware that mothers did not need to be completely reclined to enable LBBF, i.e., that it can be done with the mother’s body only slightly reclined at a 65° angle. The vast majority (over 85%) did not believe it could be done on an upright chair and felt it was a position best suited to a bed or sofa. Less than a third (29%) believed it is a position that could be used in public. There were some misunderstandings about the possibility of using LBBF when mother and baby were fully clothed, reinforcing the belief that LBBF should be reserved for skin-to-skin contact in the labour ward or on the postnatal ward in hospital. Thus, the nuances of LBBF such as the flexibility of the position were not always appreciated, making it difficult for a midwife to promote its use beyond the early days, when a mother was in company or out and about in public.

Over one-third of respondents did not recognise that LBBF has the potential to reduce breast problems, such as sore or cracked nipples, engorgement, and mastitis [6, 8, 9]—main reasons mothers give for early breastfeeding cessation [36, 37]. Greater knowledge about LBBF for midwives therefore has the potential to increase exclusive breastfeeding on discharge. Mothers post Caesarean section or who may have more challenging anatomy could receive more support from midwives who were confident in the knowledge that LBBF is suitable for them and recognise how it can be adapted for their specific situations.

Respondents frequently mentioned concerns about mothers’ anxiety, lack of confidence, hesitation to use a ‘new’ position and lack of awareness around LBBF as barriers to midwives suggesting it. New mothers tend to have heightened anxiety and lack confidence about many aspects of life with a newborn [35, 38] and infant feeding is no exception. With greater knowledge of LBBF midwives would also gain greater confidence in educating mothers about the position and addressing their concerns. This would help midwives to explain to mothers how using a LBBF position has the potential to benefit them and their babies.

Midwives and student midwives with more exposure to LBBF were much more likely to suggest it. This may be through personal experience of breastfeeding in this position, self-directed study from their own reading or attending conference sessions or from working as a lactation consultant. Attending training specifically about LBBF greatly strengthened its practice. Nearly half of all respondents who have not had this training report that they would like to. It is challenging for midwives to have the confidence and knowledge to suggest this position to help establish breastfeeding when the majority (85%) have not had specific education about how and why LBBF position works.

From the response to the open question, participants in this study appeared to rely on their traditional practices of ensuring the baby latches on with professional ‘hands-on’ help rather than supporting the innate abilities of mothers and babies.

There is much research to show that using a hands-on approach, where their breasts are held, is disliked by women [34, 39–43] and can have negative consequences for breastfeeding. The practice of attaching the infant to the breast with a grip round the infant’s neck/head and a grip around the mother’s breast/nipple can block the infant’s inborn instincts and is suggested as an underlying factor for infants’ difficulties with latching on [44].

Midwives’ traditional hands on approach to breastfeeding has also been found to lead to a pretence of mothers who appear to be coping in order to avoid seeking further help from midwives when it is needed [34, 39]. As midwives can be the key motivators for early and sustained breastfeeding [2] they are pivotal in educating mothers who may have little support once they are discharged [23]. It is vital therefore that midwives are aware of possible discomfort experienced by women when a ‘hands-on’ approach is used and how to ensure that mothers feel comfortable calling on them for further assistance.

Until very recently midwifery education has encouraged teaching mothers to breastfeed in upright positions [45]. Many respondents recognised that reluctance to use LBBF stemmed from these historic cultural teaching practices which rely on teaching methods based on formula feeding [32]. The seminal textbook for midwifery for over 60 years, Myles Textbook for Midwives [31] has for the first time included LBBF in its latest 2020 edition. This is alongside the more familiar upright positions, which it refers to as “classical breastfeeding positions” [46]. Respondents in this study were candid in admitting that it is hard to break old habits and felt more comfortable to continue to use the same techniques.

For midwives who are used to positions that emphasise health professionals as experts positioning babies, helping to place the breast into the baby’s mouth, the prone position of the baby using gravity to latch on in a LBBF position may seem counterintuitive. Education on the safety of the mother’s breastfeeding position, ensuring positional stability for the infant, and understanding primitive neonatal reflexes is vital in addressing concerns raised by midwives.

The influence of specific education for midwives on using a LBBF position for mothers and babies is further highlighted when reviewing the data (see Additional file 2).

Midwives and student midwives who had specific training about LBBF demonstrated more knowledge and reported more success in helping mothers and babies to breastfeed. Education about LBBF nearly doubled the reported confidence levels for midwives and students. The results of this study clearly show that education significantly enhanced both confidence and adoption of LBBF in practice.

Over the last decade in Ireland, rising childbirth interventions and caesarean section rates [1] have hugely increased the workload of the postnatal midwife [47]. Combined with understaffing and early discharge, these factors contribute to difficulties establishing breastfeeding [22, 48, 49]. Staffing issues in midwifery, heavy caseloads and lack of time to spend with women in the postnatal period are widely acknowledged [22, 34, 50, 51]. These were all issues which respondents in this study identified as barriers to helping women breastfeed.

Considering the ‘hands-off’ nature of a LBBF position, lack of detailed instructions needed and fewer breastfeeding problems when using this position, greater use of LBBF has the potential to reduce midwives’ workload. Encouraging LBBF empowers women to rely on their own skills, fostering increased confidence and autonomy while reducing the need for professional assistance. Any intervention that helps initiate and sustain breastfeeding is a priority for researchers, midwives, and policy makers [26, 27].

### Limitations of the study

The overall response rate of 40.8%, while not high is considered more than satisfactory [52] especially when requiring busy midwives to fill out a questionnaire. It was also dependent on the interest of link midwives to distribute and collect the survey as well as their time to do so. As participation was voluntary, respondents may have had a greater interest than non-respondents in breastfeeding and may therefore have even more awareness and understanding of LBBF than a random sample of midwives.

The results are also dependent on the information volunteered from the participants. Further research such as an observational qualitative study could provide more in-depth information. It would be important to include health care assistants, nursery nurses and NICU staff in future studies as they can be very influential in the early postnatal period when mothers are establishing breastfeeding in hospital.

Ongoing research is required to establish the effectiveness of LBBF education to determine any effects it has on midwifery practice and if this in turn is successful in reducing problems and increasing mothers’ comfort and confidence to helping maintain breastfeeding.

## Conclusions and recommendations for practice

This is the first study carried out in the Republic of Ireland to explore midwives’ and student midwives’ knowledge, attitudes, and practices of using a LBBF position. The findings point to a high awareness of LBBF, but very low use of the position in practice. It also highlights the challenges of implementing LBBF effectively in hospital settings. The study emphasises the significance of additional education about the physiological foundations of LBBF for both undergraduate and ongoing midwifery training to increase its use in practice.

Improving breastfeeding initiation and duration in Ireland is a multifactorial and complex issue. While LBBF may not be a ‘catch-all’ remedy for every breastfeeding situation, experts agree that it should be the first position to offer before other alternative positions. Enhancing midwives’ understanding regarding LBBF could help alleviate any apprehensions about adopting a more ‘hands-off’ approach, using LBBF as a primary option for women rather than a ‘fall back’ one. Although this study was specific to Ireland, it may also have relevance worldwide to cultures that continue to promote “hands on” breastfeeding for health care professionals assisting mothers in establishing breastfeeding.

### Supplementary Information

**Supplementary material 1.**

**Supplementary material 2.**

## Data Availability

The datasets used and/or analysed during the current study are available from the corresponding author on reasonable request.
