# Supplementary material for: Laid-back breastfeeding: knowledge, attitudes and practices of midwives and student midwives in Ireland
Source: Int Breastfeed J. 2024 Feb 19;19:13. doi: 10.1186/s13006-024-00619-y (PMC10877745; doi:10.1186/s13006-024-00619-y)
Supplement: Supplementary file 1 — Supplementary material 1. [file 13006_2024_619_MOESM1_ESM.pdf]

Additional file 1

Laid-back Breastfeeding Questionnaire for Midwives and Student Midwives in Ireland

Thank you for agreeing to complete the survey

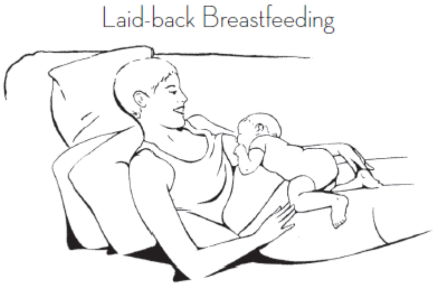

*Image Credit: La Leche League International

Laid-back breastfeeding is a baby-driven approach to positioning. The mother leans back into a semi-reclined position and the baby rests tummy down, hands free on her body with the mother’s arms acting as guardrails. This survey aims to explore what midwives and student midwives in Ireland know and think about this position and if/how they use it in their practice. There are 16 short questions to complete, which should take about 5 minutes.

Please tick appropriate box

1. What is your title?
    a. Staff midwife _____
    b. Clinical Midwife Manager _____
    c. Student midwife ______
    d. Other (please specify) __________________________

2. By which educational route did you become/are you becoming a midwife? Please select one.
    a. Direct entry - BSc (Hons) in Midwifery degree _____
    b. Post General Nursing _____

3. a. How many years have you been a qualified midwife?
    a. 0-3 years ____
    b. 4-6 years ____
    c. 7-9 years ____
    d. 10+ years ____

OR

b. What stage of your programme have you just completed?
   a. 1st Year _____
   b. 2nd Year _____
   c. 3rd Year _____
   d. I am in my Internship _____
   e. I am a Higher Diploma student _____

4. Are you a registered lactation consultant?
   a. Yes____
   b. No_____
   c. I am working towards certification_____

5. Where do you currently work? OR Where was your most recent placement? Please tick
   a. *************** Hospital _____
   b. *************** Hospital _____
   c. *************** Hospital _____
   d. Prefer not to say _____
   e. Other (please specify) ________________________________

6. Although various breastfeeding positions may be suggested depending on individual assessment, when helping mothers establish breastfeeding what is <u>the most frequent position</u> that you tend to discuss? (Tick one only)
   a. Cradle hold _____

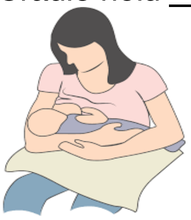

   b. Cross cradle hold _____

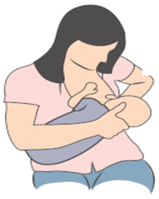

   c. Side lying _____

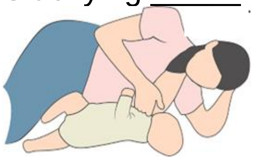

   d. Rugby hold _____

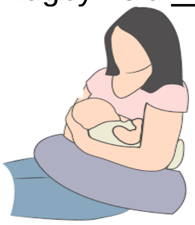

   e. Laid-back position ___

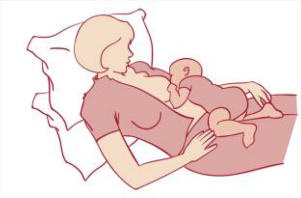

   f. Other (please specify)

   ____

   ____

   ____

7. Are you aware of the laid-back breastfeeding position as a way to help mothers to breastfeed their babies? Yes____ No*____

***If you have ticked no you have finished the questionnaire. Thank you.** 😊

8. Where did you hear or learn about laid-back breastfeeding?
   (tick as many as apply)
   a. In my midwifery education _____
   b. From other midwives _____
   c. From lactation consultants _____
   d. Self-directed study (i.e. reading, courses in lactation, conferences) ___
   e. Breastfeeding support groups (La Leche League, Cuidiú Friends of BF) ___
   f. Observing mothers and babies _____
   g. Personal experience of breastfeeding _____
   h. Other (please specify) ____

9. Have you ever had training/lectures specifically on laid-back breastfeeding, i.e. discussing positional stability for infants and the 20 primitive neonatal reflexes that this position activates?
   a. Yes, and I found it helpful for my practice _____
   b. Yes, but I didn't find it useful _____
   c. No _____
   d. No, but I would like to _____
   e. I am not sure _____

10. How often do you use a laid-back position when helping mothers?
    a. Never _____
    b. Often _____
    c. Occasionally_____
    d. Always _____

11. Which of the following best describes the scenario where you would suggest laid-back breastfeeding? (Choose one)
    a. It's my "go-to" position, to encourage mothers' own skills first_____
    b. Only when other positions do not seem helpful _____
    c. I only tend to suggest it immediately after birth with skin to skin contact to encourage the first breastfeed____
    d. As an addition to other positions _____
    e. I have never helped a mother use this position for breastfeeding _____
    f. Other ____

12. If you have helped mothers using laid-back breastfeeding how successful has it been overall?
    a. Not at all – The baby rarely latches in this position _____
    b. Somewhat – But I have to attach the baby to the breast _____
    c. Mostly – I only make a few adjustments _____
    d. Nearly always – I only need to offer encouragement ____
    e. I have not helped a mother using this position_____

13. Laid-back breastfeeding is a position that can be done (tick all that apply):
    a. In bed _____
    b. In an armchair _____
    c. In an upright/kitchen chair _________
    d. On a sofa _______________________
    e. In public ________________________

14. How confident do you feel using the laid-back breastfeeding position with mothers? (circle one)

•	•	•	•	•	•
Very	Somewhat	Neutral	Not very	Not at all	Not applicable

15. Select any of the following statements you believe to be TRUE. Laid-back breastfeeding can (Tick all that apply)

    - Be done with mother and baby fully clothed _____
    - Ensure that gravity helps to keep the baby on the mother's body reducing the need for the mother to support the baby _____
    - Reduce breast problems, such as sore nipples, cracked nipples, engorgement, and mastitis _____
    - Result in greater infant weight gain _____
    - Reduce the prescriptive pattern of *putting* the baby to the breast and allows the baby to 'head bob' and self-attach _____
    - Be done with the mother only slightly reclined at a 65° angle (with 90° being completely upright) _____
    - Reduce back, neck, and shoulder pain for the midwife from bending over to assist with latching by using a 'hands off' approach _____

16. Do you think there are any barriers that prevent a midwife from using a laid-back breastfeeding position with mothers? If so, please state.

____

____

____

Thank you so much for taking the time to complete this questionnaire!
Margaret McGuigan, 3rd year Student Midwife, DkIT
