# Supplementary material for: Laid-back breastfeeding: knowledge, attitudes and practices of midwives and student midwives in Ireland
Source: Int Breastfeed J. 2024 Feb 19;19:13. doi: 10.1186/s13006-024-00619-y (PMC10877745; doi:10.1186/s13006-024-00619-y)
Supplement: Supplementary file 2 — Supplementary material 2. [file 13006_2024_619_MOESM2_ESM.pdf]

Additional file 2: Specific training in laid-back breastfeeding and the impact of that on midwives and student midwives suggesting it.

		Have you ever attended training, lectures, or conference sessions specifically on laid-back breastfeeding, i.e., discussing positional stability for infants and the 20 primitive neonatal reflexes that this position activates?					Total
		Yes, and I found it helpful for my practice	Yes, but I did not find it useful	No	No, but I would like to	I am not sure	
Which of the following best describes the scenario where you would suggest laid-back breastfeeding?	It's my "go-to" position, to encourage mothers' own skills first	8	0	0	2	1	11
		21.6%	0.0%	0.0%	2.2%	16.7%	5.7%
	Only when other positions do not seem helpful	4	0	12	17	1	34
		10.8%	0.0%	21.1%	18.7%	16.7%	17.7%
	I only tend to suggest it immediately after birth with skin to skin contact to encourage the first breastfeed	4	1	8	15	0	28
		10.8%	100.0%	14.0%	16.5%	0.0%	14.6%
	As an addition to other positions	20	0	23	38	2	83
		54.1%	0.0%	40.4%	41.8%	33.3%	43.2%
	I have never helped a mother use this position for breastfeeding	1	0	14	19	2	36
		2.7%	0.0%	24.6%	20.9%	33.3%	18.8%
Total		37	1	57	91	6	192
		100.0%	100.0%	100.0%	100.0%	100.0%	100.0%
